# TUBB Variants Underlying Different Phenotypes Result in Altered Vesicle Trafficking and Microtubule Dynamics

**DOI:** 10.3390/ijms21041385

**Published:** 2020-02-18

**Authors:** Antonella Sferra, Stefania Petrini, Emanuele Bellacchio, Francesco Nicita, Francesco Scibelli, Maria Lisa Dentici, Paolo Alfieri, Gianluca Cestra, Enrico Silvio Bertini, Ginevra Zanni

**Affiliations:** 1 Unit of Neuromuscular and Neurodegenerative Disorders, Department Neurosciences, Bambino Gesù Children’s Hospital, IRCCS, 00146 Rome, Italy; francesco.nicita@opbg.net (F.N.); enricosilvio.bertini@opbg.net (E.S.B.); 2 Confocal Microscopy Core Facility, Research Laboratories, Ospedale Pediatrico Bambino Gesù, 00146 Rome, Italy; stefania.petrini@opbg.net; 3 Department of Research Laboratories, Bambino Gesù Children’s Hospital, 00146 Rome, Italy; emanuele.bellacchio@opbg.net; 4 Unit of Child Neuropsychiatry, Department of Neurosciences, Bambino Gesù Children’s Hospital, 00165 Rome, Italy; francesco.scibelli@opbg.net (F.S.); paolo.alfieri@opbg.net (P.A.); 5 Unit of Medical Genetics, Bambino Gesù Children’s Hospital, IRCCS, 00146 Rome, Italy; marialisa.dentici@opbg.net; 6 Institute of Molecular Biology and Pathology (IBPM), National Research Council (CNR) and University of Rome “Sapienza”, Department of Biology and Biotechnology, 00185 Rome, Italy; gianluca.cestra@uniroma1.it

**Keywords:** TUBB, tubulinopathy, microtubule dynamics, EGF transport

## Abstract

Tubulinopathies are rare neurological disorders caused by alterations in tubulin structure and function, giving rise to a wide range of brain abnormalities involving neuronal proliferation, migration, differentiation and axon guidance. TUBB is one of the ten β-tubulin encoding genes present in the human genome and is broadly expressed in the developing central nervous system and the skin. Mutations in TUBB are responsible for two distinct pathological conditions: the first is characterized by microcephaly and complex structural brain malformations and the second, also known as “circumferential skin creases Kunze type” (CSC-KT), is associated to neurological features, excess skin folding and growth retardation. We used a combination of immunocytochemical and cellular approaches to explore, on patients’ derived fibroblasts, the functional consequences of two TUBB variants: the novel mutation (p.N52S), associated with basal ganglia and cerebellar dysgenesis, and the previously reported variant (p.M73T), linked to microcephaly, corpus callosum agenesis and CSC-KT skin phenotype. Our results demonstrate that these variants impair microtubule (MT) function and dynamics. Most importantly, our studies show an altered epidermal growth factor (EGF) and transferrin (Tf) intracellular vesicle trafficking in both patients’ fibroblasts, suggesting a specific role of TUBB in MT-dependent vesicular transport.

## 1. Introduction

Complex brain malformations associated with dominant or de novo missense mutations in tubulin genes TUBA1A, TUBB2B, TUBB3, TUBB or TUBG1 are commonly referred to as “tubulinopathies” [1,2,3]. In line with the role of microtubule (MT) cytoskeleton in neurogenesis, neuronal migration, differentiation and axon guidance [4,5,6,7,8,9,10], mutations in tubulin genes can give rise to a broad spectrum of cortical and subcortical malformations. Cortical anomalies include lissencephaly, polymicrogyria, cortical dysplasia or cortical gyral simplification. Subcortical anomalies are linked to corpus callosum, hindbrain and basal ganglia. Further clinical features include microcephaly, neurodevelopmental delay and epilepsy. Specific phenotypes may be more frequently associated with mutations of particular isoforms (e.g., TUBA1A to lissencephaly or TUBB2B to polymicrogyria) [11,12,13], and at the single gene level, distinct mutations can cause either polymicrogyria or axon guidance disorders (e.g., in TUBB3) [14] depending on the variant’s impact on the dynamic properties of the cytoskeleton.

In mammals there are at least ten β-tubulin and twelve α-tubulin isotypes, possessing specific tissue and developmental distributions [15].

The complexity of the so called ‘tubulin code’ is expressed through tubulin isotype composition and chemical diversity processes, such as the interaction with microtubules-associated proteins (MAPs) and tubulin post-translational modification enzymes (PTMs), tuning microtubule dynamics, neuronal polarity, cell motility and intracellular trafficking [16,17]. Neuronal-specific TUBB3 and TUBB4 are responsible for the efficient transport of N-cadherin to the cell membrane, and their upregulation enhance cell migration during the endothelial-mesenchymal transition [18].

Class I β-tubulin TUBB (also known as TUBB5) is highly expressed in neuronal progenitors, in postmitotic neurons during fetal brain development [19] and in primary human fibroblasts [20]. Patients with TUBB mutations exhibit neurological features including microcephaly, dysgenesis of the cerebellum and the basal ganglia, agenesis of the corpus callosum and congenital symmetrical circumferential skin creases (CSC-KT) [21,22]. The variability of patient phenotypes underscores the possibility, seen in other tubulinopathies, that specific TUBB mutations may have different impacts on MT functions. However, only few studies have explored the molecular and cellular consequences of TUBB mutations [23,24]. We performed comparative functional studies using primary fibroblasts of two patients presenting different TUBB-related clinical features: pure neurological or CSC-KT phenotype. The analysis of TUBB-mutated fibroblasts showed multiple defects in MT polymerization, vesicle trafficking and cell migration.

## 2. Results

### 2.1. TUBB Gene Mutation Analysis

**Figure 1:**
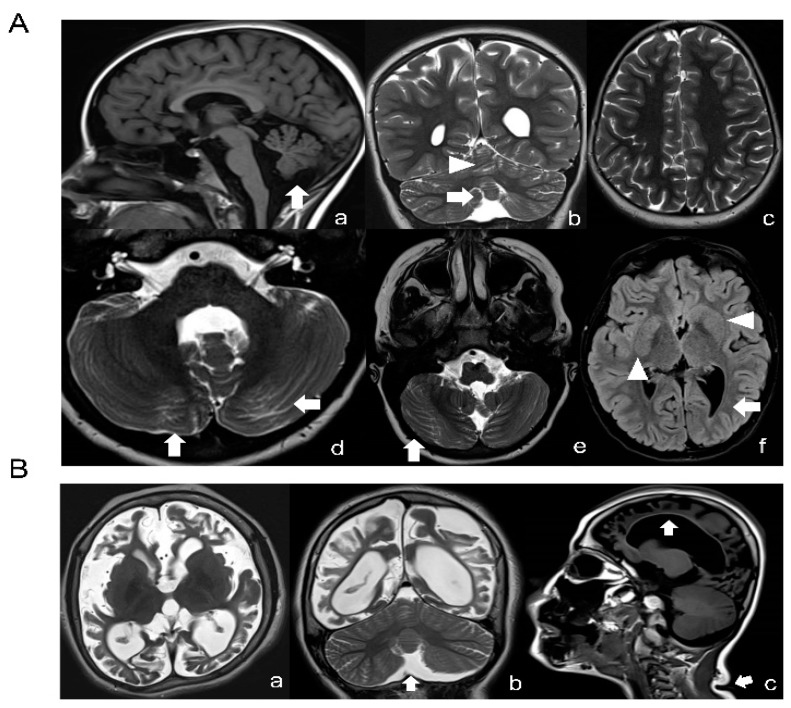
Neuroimaging studies of the patients. (**A**) Brain MRI of patient 1 (N52S) at the age of 10: T1-weighted (**a**), T2-weighted (**b**–**e**) and FLAIR (**f**) images revealing a normal corpus callosum and lower vermis hypoplasia with an increased fourth ventricle (arrow in **a** and **b**) and absence of cortical dysplasia (**c**), but presence of dysplasia of the cerebellar folia in both vermis and hemispheres (arrows in **a**, **b**, **d** and **e**), basal ganglia anomalies (asymmetric and abnormal shaped lenticular and caudate nuclei, arrowheads in **f**) and lateral ventricle asymmetry with left prevalence and left occipital with matter reduction (arrow in **f**). (**B**) Brain MRI of patient 2 (M73T) at the age of 9: T2-weighted (**a**,**b**) and T1-weighted (**c**) images; severe cortical atrophy with absence of subcortical white matter is seen (**a**–**c**), together with ex vacuo dilatation of the lateral ventricles, increased subarachnoid spaces and a thin corpus callosum (arrow in **c**). Mild hypoplasia of the lower cerebellar vermis (arrow in **b**) without dysplasia. The basal ganglia seem to be relatively spared. Note, moreover, a crease in the posterior neck (arrow in **c**).

A novel missense variant c.155A > G (p.N52S) in TUBB (NM_178014) was detected by targeted next-generation sequencing in patient 1, who presented dysmorphic basal ganglia and cerebellar dysgenesis with normal head circumference and corpus callosum. A segregation analysis demonstrated a de novo origin of the mutation. The variant is not reported in public databases (i.e., dbSNP146, 1000 Genomes, ExAC and GnomAD). The missense variant c.218T > C (p.M73T) identified in patient 2, presenting microcephaly, seizures and CSC-KT skin phenotype, has been reported previously [25]. A detailed description of the clinical features of both patients are provided in Tables S1 and S2. Neuroimaging studies of patient 1 revealed dysplasia of the cerebellar folia, hypoplasia of the lower cerebellar vermis with enlarged fourth ventricle, lateral ventricle asymmetry and abnormally shaped lenticular and caudate nuclei (Figure 1A). In patient 2, the basal ganglia were normal but a marked atrophy of the cerebral hemispheres with increased ventricular and periencephalic liquoral spaces, severe corpus callosum hypoplasia, lower vermis hypoplasia and a left frontal parietal arachnoid cyst were detected (Figure 1B).

### 2.2. N52S and M73T Substitutions Affect Microtubule Dynamics In Vitro

**Figure 2:**
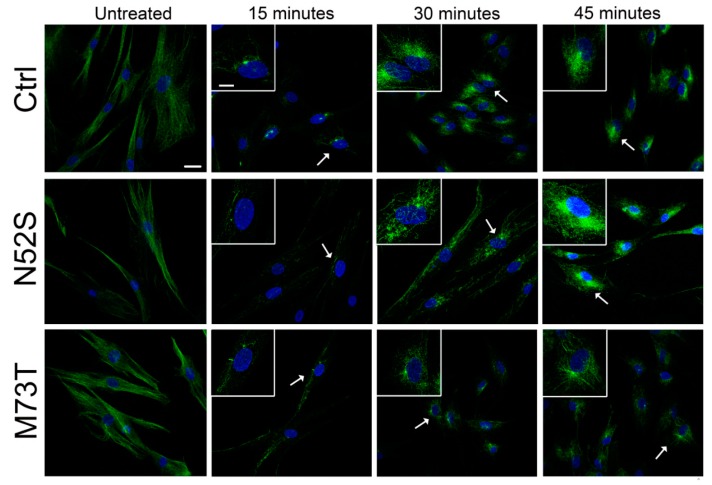
N52S and M73T variants affect MT dynamics after nocodazole treatment. Patients’ fibroblasts were treated with nocodazole. After the substance was washed out, they were analyzed by immunofluorescence experiments to visualize the rate of microtubule growth. The microtubules were labeled with β-tubulin (green) and nuclei were labeled with Hoechst (blue). The scale bar represents 25 μm. The inserts are magnifications of the cells indicated by the arrows. The scale bar represents 10 μm.

**Figure 3:**
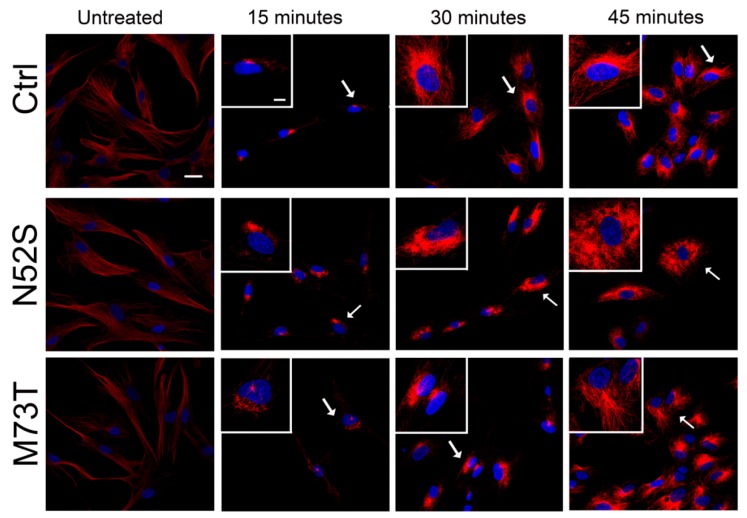
N52S and M73T variants affect α-tubulin incorporation into MTs after nocodazole treatment. Patients’ fibroblasts were treated with nocodazole. After the substance was washed out, they were analyzed at different time points by immunofluorescence experiments to visualize the rate of microtubule growth. The microtubules were stained with α-tubulin (red) and the nuclei were stained with Hoechst (blue). The scale bar represents 10 μm. The inserts are magnifications of the cells indicated by the arrows. The scale bar represents 10 μm.

We explored the effect of N52S and M73T mutations on MT dynamics in vitro, evaluating the ability of MTs to reassemble after nocodazole treatment. Primary fibroblasts of healthy subjects and patients were treated with the MT disrupting drug nocodazole. After its wash-out, MT repolymerization was analyzed by immunofluorescence in time course experiments. The immunostaining analysis of human primary fibroblasts, using anti α- and β-tubulin antibodies, detected alterations of MT dynamics in both the N52S and M73T cells (Figure 2 and Figure 3). In N52S fibroblasts, differently from control cells in which MTs form an extended and defined network during repolymerization, MTs appeared strongly disorganized, with the accumulation of tangled structures clearly visible after 30 and 45 min of recovery (Figure 2 and Figure 3). In M73T fibroblasts, the MT polymerization rate was delayed, and only a slower integration of α- and β-tubulin into MTs was observed after 30 min of recovery (Figure 2 and Figure 3). These data suggest that, whereas M73T substitution affects the MT polymerization rate, N52S mutation impairs MT spatial organization during polymerization.

### 2.3. EGF and Tf Transport Are Impaired in M73T and N52S Mutant Fibroblasts

Considering the pivotal role of MTs in intracellular vesicle trafficking [26], we explored the impact of N52S and M73T mutations on the intracellular transport of endocytic vesicles.

We analyzed the transport of fluorescently labeled EGF and Tf, which relies on MT tracks and can be easily evaluated analyzing their fluorescence, representing a useful tool to assess vesicle transition and localization. Patient and control fibroblasts were serum-starved and then incubated with fluorescently-labeled EGF. The intracellular localization of EGF-positive postendocytic vesicles was analyzed 20 min after internalization.

**Figure 4:**
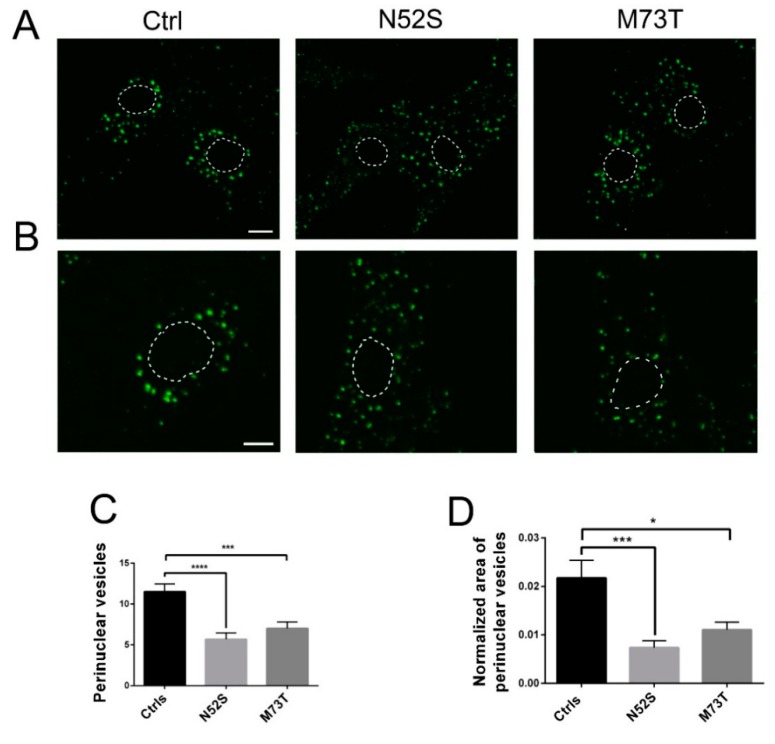
Confocal analysis of EGF transport in control and mutated fibroblasts. (**A**) Patients and control fibroblasts were incubated with Alexa fluor 488-labeled EGF and, after 20 min of internalization, were analyzed by confocal analysis to visualize the EGF localization. The nuclear region is delimited by the dotted circle. The scale bar represents 10 μm. (**B**) Higher magnifications. The nuclear region is delimited by the dotted circle. The scale bar represents 10 μm. (**C**) Quantification of EGF vesicles in the perinuclear region of patients and control fibroblasts after 20 min of internalization. (**D**) Quantification of the mean area of EGF vesicles (total area of EGF spots/area of the perinuclear region). Student’s *t*-test, * *p* ≤ 0.05, *** *p* ≤ 0.0005, ***** *p* ≤ 0.0001.

As shown in Figure 4, in control fibroblasts fluorescence-labeled EGF accumulated in the perinuclear vesicles; conversely, in N52S and M73T mutated cells the perinuclear accumulation of EGF was significantly impaired. EGF vesicle trafficking was particularly compromised in N52S mutated fibroblasts, where EGF positive puncta were dispersed through the cytoplasm (Figure 4A,B).

To gain more quantitative insight into EGF trafficking, we evaluated the number and the normalized area of EGF spots in the perinuclear region. The number and the size of EGF spots were significantly decreased in both patients’ cells. In particular, they were mainly impaired in N52S fibroblasts (Figure 4C,D), suggesting that this mutation has a stronger impact on vesicular transport.

**Figure 5:**
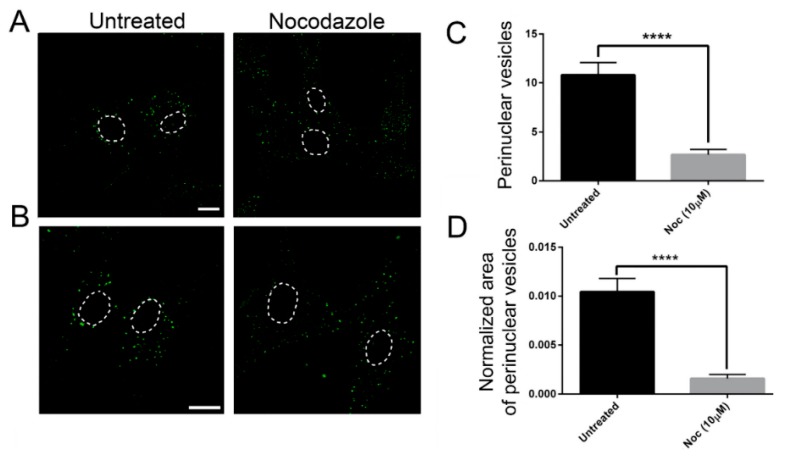
The effect on EGF transport is similar in nocodazole-treated control fibroblasts and untreated fibroblasts carrying TUBB mutations (**A**,**B**) Control fibroblasts were incubated with Alexa fluor 488-labeled EGF with or without nocodazole (Noc). After 20 min of internalization, they were analyzed by confocal analysis to visualize the EGF localization. The nuclear region is delimited by the dotted circle. The scale bar represents 10 μm. (B) Higher magnifications. The nuclear region is delimited by the dotted circle. The scale bar represents 10 μm. (**C**) Quantification of EGF vesicles in the perinuclear region of control fibroblasts after 20 min of internalization. (**D**) Quantification of the mean area of EGF vesicles (total area of EGF spots/area of the perinuclear region). Student’s *t*-test, **** *p* ≤ 0.0001.

In order to confirm the role of MTs on EGF postendocytic vesicle trafficking, we also analyzed the effects of nocodazole treatment on this transport. As shown in Figure 5, control cells treated with nocodazole showed a prominent reduction of perinuclear EGF positive puncta (Figure 5), as previously observed in the patients’ cells. These data strongly suggest that defective dynamics of MTs either due to nocodazole or to TUBB mutations have a similar impact on EGF vesicle trafficking.

**Figure 6:**
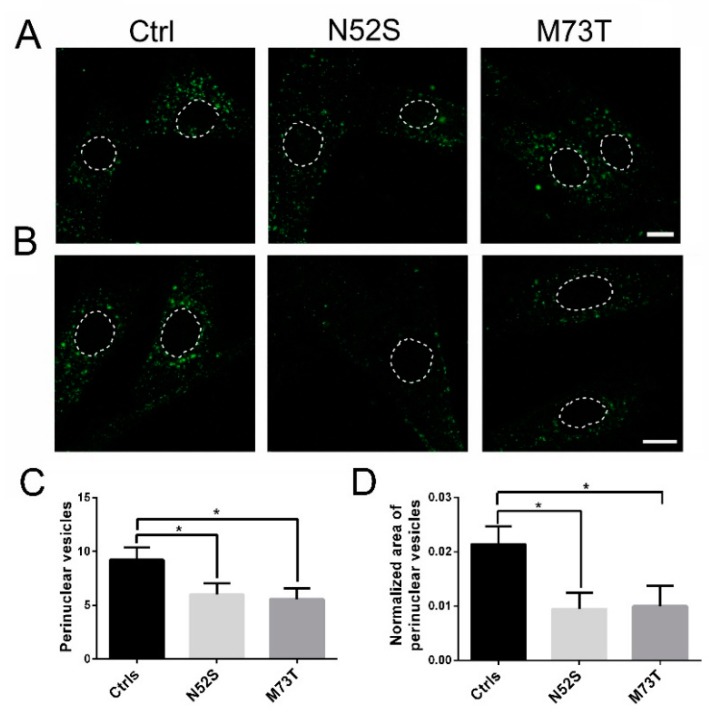
Confocal analysis of Tf transport in control and mutated fibroblasts. (**A**,**B**) Patients and control fibroblasts were incubated with Alexa fluor 488-labeled Tf and, after 20 min of internalization, were analyzed by confocal analysis to visualize the Tf localization. The nuclear region is delimited by the dotted circle. The scale bar represents 10 μm. (**B**) Higher magnifications. The nuclear region is delimited by the dotted circle. The scale bar represents 10 μm. (**C**) Quantification of Tf vesicles in the perinuclear region of patients and control fibroblasts after 20 min of internalization. (**D**) Quantification of the mean area of Tf vesicles (total area of Tf spots/area of the perinuclear region). Student’s *t*-test, * *p* ≤ 0.05.

To further confirm the impact of TUBB mutations on the intracellular vesicle transport, we evaluated the MT-mediated trafficking of vesicles carrying Tf. Interestingly, we observed a similar reduction of Tf perinuclear puncta in the patients’ cells (Figure 6).

### 2.4. N52S and M73T Variants Affect Fibroblasts’ Migration

**Figure 7:**
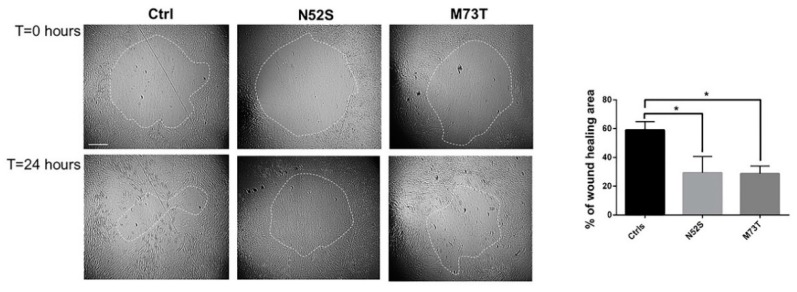
Study of cell motility in control and mutated fibroblasts. Cell migration ability of patient fibroblasts as compared with age-matched control cells, evaluated by in vitro migration assay. On the left, representative phase-contrast photographs of the gap closure taken at the indicated time intervals are shown. The gap areas are delimited by the dotted circles. The scale bar represents 400 μm. The results represent the mean ± SEM of three independent experiments, each performed at least in triplicate (total evaluated gaps *n* = 36 for control fibroblasts, using two different lines; *n* = 14 for N52S mutated fibroblasts, *n* = 16 for p.M73T mutated fibroblasts). Student’s *t*-test, * *p* ≤ 0.005.

Several pieces of evidence have shown that MTs operate synergistically with actin to establish and maintain the spatial/temporal coordination of cell migration [27]. To explore the effect of N52S and M73T mutations on cell motility, we performed a silicon stopper based cell migration assay. As shown in Figure 7, the rates of closure of the central zone after 24 hours was reduced in both mutated fibroblasts as compared with cells from age-matched healthy subjects. The migration rate was affected with similar severity in both patients’ cells, in which the rate of wound healing, 24 h after the stopper removal, was 28.7% and 29.4% for p.M73T and N52S mutated fibroblasts, respectively, differently from control fibroblasts, in which the rate of wound healing was 57.9 % (Figure 7).

### 2.5. Structural Modeling of TUBB Variants

**Figure 8:**
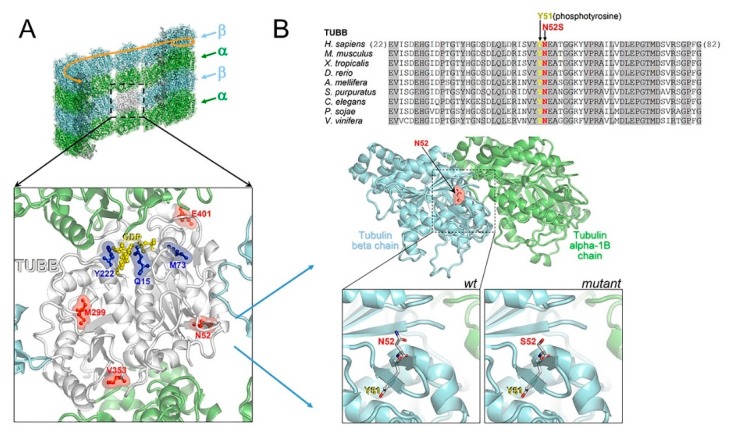
Comparative mapping of TUBB mutations and structural modeling of the N52S mutation. (**A**) Multiple sequence alignment of tubulin beta chain proteins from different organisms around the site affected by the N52S amino acid change (invariant residues are grayed), and homology model of the human TUBB (N52 is indicated) in the complex with tubulin alpha-1B. In the wildtype inset, the contiguous phosphorylatable Y51 is shown. The mutant inset represents a modeled N52S amino acid replacement. (**B**) TUBB model (white ribbons) overlaid onto a microtubule, and amino acid sites affected by “neurological type” (red) and “Kunze type” (blue) variants. Of note, all “Kunze type” variants map near the GTP/ guanosine-5’-diphosphate (GDP) binding site (the bound GDP molecule is shown in yellow).

In an in silico structural 3D model, we mapped all reported CSC-KT-related mutations (p.Y222F, p.Q15K and p.M73T) and variants associated with the pure neurological phenotype (p.M299V, p.V353I, p.E401K, N52S) on a TUBB-α tubulin heterodimer (Figure 8A). Although TUBB mutations causing CSC-KT affect residues not contiguous in the primary sequence, the in silico model of the heterodimeric complex showed that all mutations were clustered near the guanosine-5’-triphosphate (GTP) binding site, whereas the other variants, including N52S, were located outside the GTP binding domain, in structural and functionally distinct regions (Figure 8A). Specifically, N52 is an amino acidic residue highly conserved among TUBB orthologs and paralogs and positioned close to the α-β tubulin heterodimer and the lateral protofilament interface (Figure 8B). N52 is also contiguous to Y51, which has been reported to undergo phosphorylation [28]. Therefore, although the N52S substitution has the potential to compromise α-β tubulin interaction and the dynamic behavior of microtubules, we cannot exclude that this change could disrupt the consensus sequence motif for the phosphorylation of Y51, affecting the participation of the site in post-translational modifications and interactions with MAPs.

## 3. Discussion

In this study we have explored the cellular defects associated with two different TUBB mutations underlying distinct clinical phenotypes: a neurological condition, characterized by basal ganglia and cerebellar dysgenesis, identified in a subject harboring a novel missense N52S substitution; and the CSC-KT phenotype caused by a previously reported M73T mutation [25].

Since TUBB is highly expressed in primary fibroblasts (Figure S1), the impact of these mutations was analyzed in patients’ fibroblasts that represent a suitable cell type to assess the effects of TUBB mutations on MT functions and behavior.

We first explored the effect of N52S and M73T mutations on MT dynamics in vitro, evaluating the ability of MTs to reassemble after nocodazole treatment. Immunofluorescence analysis showed slower MT repolymerization in p.M73T mutated fibroblasts, consistent with the hypothesis that M73T substitution, being located in a critical region near the GTP binding domain, influences the ability of MTs to polymerize (Figure 8). In N52S mutated fibroblasts, although the substitution does not affect the polymerization rate, MTs appeared strongly disorganized and arranged in tangled filaments, visible during the late stages of growth, suggesting that this mutation may affect primarily MT spatial organization. Consistently with this observation, the N52 residue was far from the GTP binding pocket and in close proximity to the lateral protofilament interface that may be directly involved in the regulation of MT organization.

Further, we investigated the effect of TUBB mutations on EGF and Tf postendocytic vesicle transport and cell movement, processes that rely both on the tight regulation of MTs and on other cytoskeleton network components.

MTs participate in the spatial organization of endocytic vesicles [29,30], and it is well established that all types of endosomes actively move along MTs [31]. Moreover, MTs, together with actin filaments and motor proteins, provide the mechanistic force to deform membranes, thereby facilitating cargo sorting [26]. Disruption of MTs strongly affects endosome motility and causes the dispersal of both endosomes and lysosomes throughout the cytoplasm [32,33,34,35].

Both EGF and Tf vesicle transport were impaired in patients’ fibroblasts that showed a significant reduction of EGF and Tf positive perinuclear puncta localization after 20 min of internalization. Moreover, EGF and Tf vesicle trafficking were particularly altered in N52S mutated fibroblasts, where their spots puncta were mostly dispersed through the cytoplasm, suggesting that this mutation had a more severe impact on their postendocytic transport.

In M73T mutated cells, EGF and Tf spots showed a predominant perinuclear localization. However, the number and the normalized area of EGF spots result significantly decreased. Interestingly, the prominent effects of N52S on membrane trafficking and MT organization, combined with its minor influence on the MT polymerization rate, suggests that EGF and Tf vesicle transport may be more sensitive to alterations of MT orientation.

We also analyzed the effect of nocodazole on fibroblast EGF transport. Nocodazole treatment drastically impaired EGF distribution, demonstrating that defective dynamics of MTs either due to nocodazole or to TUBB mutations have a similar impact on EGF trafficking.

These data point to a relevant role for TUBB in postendocytic transport; interestingly, the expression level of TUBB5 was found to be upregulated during the endocytosis-mediated invasion of S. agalactiae in H9C2 cells [36], and Jeon and colleagues, using a cell sorting-based functional selection of phagocytosis promoting genes, identified TUBB5 as one of the genes conferring phagocytic activity to mouse fibroblasts [37]. Moreover, vesicle trafficking, controlling the availability and spatial distribution of cargoes, influences the movement of cells [38,39], and the impairment in EGF signaling perturbs the migration of pancreatic islet cells and keratinocytes in EGF- receptor deficient mice [40,41].

Compelling evidence has shown that MTs operate synergistically with actin to establish and maintain the spatiotemporal coordination of cell migration [28,42,43]. During movement, cells first extend a leading edge, the lamellipodium, by assembling a branched network of actin filaments under the plasmamenbrane. The lamellipodium protrudes, adheres to the surface toward the direction of movement and pulls the whole body of the cell forward [44,45]. Although the protrusion of the lamellipodia is regulated by actin cytoskeleton polymerization, several works have shown that the disruption of MTs in fibroblasts affects the lamellipodia’s control of membrane ruffling formation [46]. Furthermore, recent studies showed that mutations in tubulin genes specifically affect the directional motility of cells [27]. Cell motility is determinant during brain and craniofacial development, and the positioning of postmitotic neurons and neural crest cells [47,48] into appropriate spatial relationships depends on their capacity to move and reach their final location. Defective neuronal migration arrests different types of neurons along the migratory path, resulting in gross brain malformations and cerebral structural abnormalities [49,50]. We demonstrated that both TUBB substitutions impair fibroblast motility. Accordingly, Breuss and colleagues showed that the overexpression of human TUBB mutants M299V, V353I and E401K in the brain of mice determines long-term migratory defects, and that the depletion of TUBB5 in mice alters the positioning of migrating neurons [19].

To further clarify the genotype- phenotype correlations of TUBB-related tubulinopathies, we mapped all reported mutations related to CSC-KT (p.Y222F, p.Q15K and p.M73T) and pure neurological phenotypes (p.M299V, p.V353I, p.E401K, p.N52S) in the quaternary structure of the TUBB5/α-tubulin complex. The in silico model showed that all CSC-KT mutations identified to date affect amino-acidic residues closely surrounding the GTP binding pocket, while the variants associated to neurological phenotypes in the absence of CSC-KT features are located outside the GTP domain. Therefore, the previous correlation between the N-terminal or C-terminal related TUBB mutation in the presence or absence of CSC-KT features, respectively, should be reconsidered.

Overall, our comparative analysis of fibroblast cell lines derived from patients harboring different mutations in TUBB, and presenting clinically distinct phenotypes, provides further insights into cellular defects caused by MT dysfunction underlying the phenotypic variability of tubulinopathies.

Future genomic and functional studies using neuronal models derived from patients’ cells might help further characterize TUBB-related phenotypes and target future therapies.

## 4. Materials and Methods

### 4.1. Cell Culture

Human primary fibroblasts of patients and healthy age-matched controls were grown at 37 °C in Dulbecco’s modified Eagle’s medium (high glucose formulation) supplemented with 10% fetal bovine serum (Invitrogen, Thermo Fisher Scientific, Waltham, MA and 1% penicillin-streptomycin antibiotics in a 5% CO_2_ in air, humidified atmosphere. All studies were performed in accordance with the Declaration of Helsinki and IRB approval 1779_OPBG_2019 (24 September 2019). Written informed consent was obtained from all participating subjects.

### 4.2. Nocodazole Wash-out and Immunofluorescence Staining

Healthy control and patient fibroblasts were seeded on coverslips in 6-well plates (12 × 104) and, after 24 h, the microtubules were depolymerized by adding nocodazole (10 μM) to the medium and incubated at 37 °C for 30 min. Nocodazole was washed out with a warm culture medium, and fibroblasts were further incubated for 15, 30 and 45 min. Cultures were rinsed in PHEM (60 mM PIPES, 25 mM HEPES, 10 mM EGTA, 2 mM MgCl2 and 1% formaldehyde, pH 6.9) and extracted for 3 min in PHEM containing 0.2% Triton X-100 and 20M taxol, fixed, blocked (5% bovine serum albumin for 1 h) and immunostained with the indicated primary antibodies: mouse anti-α tubulin (1:500, 1 h, Sigma-Aldrich, St Louis, MO, USA) rabbit anti-β tubulin (1:500, overnight, Cell Signaling Technology, Leiden, The Netherlands).

### 4.3. Confocal Analysis

Confocal microscopy was performed on a Leica TCS-SP8X laser-scanning confocal microscope (Leica Microsystems, Mannheim, Germany) equipped with a white light laser source and a 405 nm diode laser. Sequential confocal images were acquired using a HC PLAPO CS2 40× oil-immersion objective with a 1024 × 1024 format, 400 Hz scan speed and a z-step size of 0.4 µm. The lasers’ power, beam splitters, filter settings, pinhole diameter and scan mode were the same for all examined samples of each staining.

### 4.4. EGF and Tf Internalization Assay

Control and patient fibroblasts were seeded on coverslips in 6-well plates (70 × 103) and, after 24 h, starved for 16 h. The cells were incubated for 1h in ice with Alexa fluor 488-labeled EGF (100 ng/mL; Thermofisher, Waltham, MA, USA) or Alexa fluor 488-labeled Tf (100 μg/mL; Thermofisher, Waltham, MA, USA) with or without sucrose solution (0.45 M), which prevented their internalization (negative control). After incubation with labeled EGF or Tf, the fibroblasts were washed once with ice cold Tyrode’s buffer and once with prewarmed Tyrode’s buffer and transferred to 37 °C for 20 min. After the incubation, the coverslips were washed once with ice cold Tyrode’s buffer, three times with ice cold acid buffer and once with ice cold Tyrode’s buffer and fixed with PFA 4% for 20 min.

The quantification of the perinuclear vesicles was performed using Image J (NIH, downloadable at http://rsbweb.nih.gov/ij/download.html). The image was converted into a binary image using the menu command Image > Type > 8-bit. The threshold was set to B&W. The perinuclear region was selected using the oval tool, and the number and the area of vesicles into the perinuclear region were analyzed using the command Analyze > Analyze particles. The minimum size of area was set to 50 pixels, to exclude aspecific particles.

### 4.5. Cell Migration Assay

The migration rate was evaluated using the OrisTM Cell Migration Kit (Platypus Technologies, Madison, WI, USA) following the manufacturer’s instructions. Briefly, the control and patient fibroblasts were seeded (5 × 104/well) into 96-well plates containing medical-grade silicon stoppers and incubated 3 h with 10 μg/mL mitomycin (Sigma-Aldrich, St Louis, MO, USA) to block cell proliferation. Then, the silicon stoppers were removed, and the residual gap zone was photographed with a microscope Leica DMi8 at 0 h and after 24 h. Image J (NIH, available online: http://rsbweb.nih.gov/ij/download.html) was used to calculate the percentage of cell-free area. The experiments were carried out in triplicate, and residual gap areas were expressed as a percentage of the initial corresponding scratched area.

### 4.6. Data analysis and Statistics

The statistical analysis was performed using the GRAPHPAD/Prism 7.0 Software (GraphPad Software, San Diego, CA, USA). Statistically significant differences between groups were analyzed using the Student’s *t*-test for normally distributed variables. All data are presented as mean ± standard error of the mean (SEM). Statistical significance was defined as * *p* ≤ 0.05, ** *p* ≤ 0.005, *** *p* ≤ 0.0005, **** *p* ≤ 0.0001.

### 4.7. Homology Modeling of the Human Tubulin Beta Chain

The homology modeling analysis of the human tubulin beta chain (TUBB) was made on the Protein Data Bank (PDB) with the structure 5KMG (representing the TUBB protein from pig complexed with the tubulin alpha-1B chain and a fragment of the protein regulator of cytokinesis 1) as the template. Since the site of the N52S mutation and the phosphorylatable Y51 are conserved in human and pig TUBB (the two proteins share 98% amino acid identity), the model of human TUBB was obtained by renumbering the amino acid residues of the pig TUBB protein to the residues of the human homologue. The tubulin alpha-1B chain and the guanosine-5’-diphosphate (GDP) co-crystallized with the template have been retained. The TUBB model was overlaid onto a microtubule structure (PDB 5SYE) to map the sites of variants associated to the pure neurological phenotype or to the CSC-KT skin phenotype, with respect to the other bound tubulin chains within the microtubule.

## Supplementary Materials

Supplementary materials can be found at https://www.mdpi.com/1422-0067/21/4/1385/s1.

## Abbreviations

CSCKT	Circumferential skin creases Kunze type
MT	Microtubule
EGF	Epidermal growth factor
Tf	Transferrin
MAPs	Microtubule-associated proteins
PTMs	Post-translational modifications
