# Supplementary material for: TUBB Variants Underlying Different Phenotypes Result in Altered Vesicle Trafficking and Microtubule Dynamics
Source: Int J Mol Sci. 2020 Feb 18;21(4):1385. doi: 10.3390/ijms21041385 (PMC7073044; doi:10.3390/ijms21041385)
Supplement: Supplementary file 1 [file ijms-21-01385-s001.pdf]

SUPPLEMENTAL DATA

TUBB variants underlying different phenotypes result in altered vesicle trafficking and microtubule dynamics.

Antonella Sferra [1*], Stefania Petrini[2], Emanuele Bellacchio[3], Francesco Nicita[4], Francesco Scibelli[5], Maria Lisa Dentici[6], Paolo Alfieri[7], Gianluca Cestra[8], Enrico Silvio Bertini[9] and Ginevra Zanni[10*].

Clinical Description of patient 1

The patient was assessed by using a neuropsychological protocol at Bambino Gesù Children's Hospital in Rome at the age of 13.6 years. The patient was born after an uncomplicated pregnancy at 38 weeks of gestation. Her birth weight was 2,760 KG. Nuclear magnetic resonance (NMR) has revealed cerebellar and brain stem hypoplasia. The patient was aware and compliant. The evaluation of motor and postural skills has shown an ataxia and a slight scoliotic attitude. Walking motor skills were impaired and characterized by motor clumsiness, even more acute in distress condition and/or during episode of infection. Static and dynamic balancing was preserved despite the clinical condition. The patient was negative for CGH Array test. The Body Mass Index of the patient was 16.2 (40 KG; 157 CM).

The neuropsychiatric assessment has revealed anxiety symptoms as follows: difficulty falling asleep associated with frequent awakenings, easy irritability and oppositive behaviors, prevalent within the family context. Criteria for Anxiety Disorder were not fully reached. Furthermore, the assessment has shown a slight impairment in regulation and expression of emotional states. The patient was also cared from community health service for learning difficulties.

Materials and Methods

The neuropsychological assessment has been conducted by using a battery of neuropsychological tests. Intelligence Quotient has been assessed through The Wechsler Intelligence Scale for Children III (WISC III, Wechsler 1991), a clinical and diagnostic test widely used for the evaluation of cognitive skills in children and adolescents from 6 years to 16 years and 11 months.

The Schedule for Affective Disorders and Schizophrenia for School Age Children, Present and Lifetime version (K-SADS PL, Kaufman et al. 2000) has been used to detect current and past features of psychopathological signs/psychiatric disorders according to DSM-IV criteria (4th ed; DSM-IV; American Psychiatric Association [APA], 1994). Emotional Competence has been assessed through Toronto Alexithymia Scale (TAS, Bagby et al., 1994), a self-report tool originally designed for the Alexithymia Symptoms, routinely used for the evaluation of emotional awareness. Tas consists of three subscales (Difficulties Identifying Feelings, Difficulties Describing Feelings, and Externally Oriented Thinking) and a total scale (ranging from 20 to 100). Tas have 3 score range: from 0 to 50 "non alexithymic subjects", from 51 to 60 "borderline", from 61 to 100 alexithymic subjects".

Academic skills have been evaluated through BVSCO-2 (Battery for the assessment of writing skills in Italian children between 7 and 13 years, Tressoldi et al. 2013), an Italian standardized writing battery. It has been administered a text dictation test. Motor skills were assessed using Movement Assessment Battery for Children–Second Edition (MABC–2) (Henderson et al., 2007). This test identifies motor impairments using three specific motor area composites (Manual dexterity, Aiming and Catching, Balance) and a Total Score.

Results

The patient was assessed at the age of 13.6 years (Table I). Wisc III has revealed a cognitive functioning on the low average. Concerning psychopathological features, the patient does not meet full criteria for any diagnosis on the K-SADS PL. The interview has revealed anxiety traits like frequent awakenings, easy irritability and oppositive behavior, especially with family members. Parents of the patient indicate adherence to rigid and specific routines. Moreover, the patient has reported impairment in recognizing and regulating emotional states. Regarding emotional competence, measured with TAS, the patient obtained a total borderline score, significant for Alexithymia Traits.

According to the BVSCO-2, academic skills are considered adequate for age and scholarization; qualitative observation has not revealed difficulties in graph motor skills.
Regarding M-ABC-2, the patient obtained a total score on the average limit with uneven distribution of subscales scores (“Manual dexterity” under the average; “Aiming/cathing” and “balance” on the average). To the clinical observation the patient has shown slowness of manual dexterity process in the first two items of the subtest (“turn the lite-brite”; “turn the bolts”). Furthermore, the patient has shown good abilities in throwing and grasping a tennis ball with both hands; the skill “throw a ball towards a 2,5 meters target” is preserved. The patient has also good abilities in “Zig-zag hopping on mats” and in “Walking heel to toe backward”; fair skills in balance on a table.

Table S1: Neuropsychological Assessment

**Assessment**	**Results**
Intelligence Quotient; WISC III	Total I.Q.: 86; -0.9 SD; 17.4° PC
Psychopatological features; K-SADS-PL	Criteria not reached for any diagnosis.<br>Anxiety Traits as follow: frequent awakenings, easy irritability and oppositive behaviors
Emotional competence; TAS	Total Scale: 53;<br>Difficulties Identifying Feelings: 14;<br>Difficulties Describing Feelings: 12;<br>Externally Oriented Thinking: 27.
Academic skills; BVSCO-2	Text Dictation: 1 error; -0.7 SD; >60°PC<br>Adequate performance
Motor Skills; M-ABC-2	Total Score (TC): SS 6; -1.3 SD; 9° PC;<br>Manual Dexterity (MD): SS 5; -1.6SD ; 5° PC;<br>Aiming and Catching (A&C): SS 8; -0.6 SD; 25° PC;<br>Balance (B): SS 9; -0.33 SD; 37° PC.

I.Q. = Intelligence quotient; PC = Percentile; SD = Standard Deviation; SS = Standard Score

References

Bagby, R. M., Taylor, G. J., & Parker, J. D. (1994). The twenty-item Toronto Alexithymia Scale—II. Convergent, discriminant, and concurrent validity. Journal of psychosomatic research, 38(1), 33-40.
Henderson, S. E., Sugden, D. A., & Barnett, A. L. (2007). Movement assessment battery for children-2. Harcourt Assessment.
Kaufman, J., Birmaher, B., Brent, D. A., Ryan, N. D., & Rao, U. (2000). K-sads-pl. Journal of the American Academy of Child & Adolescent Psychiatry, 39(10), 1208
Tressoldi, P. E., Cornoldi, C., & Re, A. M. (2013). BVSCO-2: Batteria Per la Valutazione Della Scrittura E Della Competenza Ortografica-2: Manuale E Materiali Per Le Prove. Giunti OS.
Wechsler, D. (1991). The Wechsler intelligence scale for children—third edition. San Antonio, TX: The Psychological Corporation.

	**Patient 1**	**Previous Mic-BA cases**	**Patient 2**	**Previous CSC-KT cases**	**Total of *TUBB5* cases**
**Phenotype**					
**CSC-KT**	-	-	1	3	4/10
**Mic-BA**	1	5	-	-	6/10
**Sex (M/F)**	F	2M, 2F (1NR)	M	2M, 1F	4F, 5M, 1NR
***De novo*/Inherited**	*de novo*	5/5 *de novo*	Inherited	3/3 *de novo*	9/10 de novo
**Cognitive delay**					
**Learning disability**	Yes	-	-	-	1/10
**Mild**	-	3/5	-	3/3	6/10
**Moderate**	-	1/5	-	0/3	1/10
**Severe**	-	1/5	Yes	0/3	2/10
**Language delay**	No	4/4 (1 NR)	Yes	2/2 (1 NR)	7/8 (2 NR)
**Motor delay**	Yes	3/4 (1 NR)	Yes	1/2 (1 NR)	6/8 (2 NR)
**Microcephaly**	No	5/5	Yes	3/3	9/10
**Other features***	No	4/5	Yes	3/3	8/10
**MRI findings**					
**Normal**	-	0/5	-	2/3	2/10
**Cortical dysgenesis**	-	1/5	-	0/3	1/10
**CC anomalies^**	-	5/5	Yes	1/3	7/10
**Dysmorphic BG**	Yes	3/5	-	0/3	4/10
**White streaks in BG**	-	2/5	-	0/3	2/10
**Cerebellar anomalies**	Yes	1/5	Yes	0/3	3/10
**Other°**	Yes	2/5	Yes	1/3	5/10
***TUBB5* mutation**					
**Missense****	N52S	5/5	M73T	3/3	10/10
**Other**	-	0/5	-	0/3	0/10

Table S2. Clinical and neuroradiological features of patients and previously reported cases of *TUBB* mutations

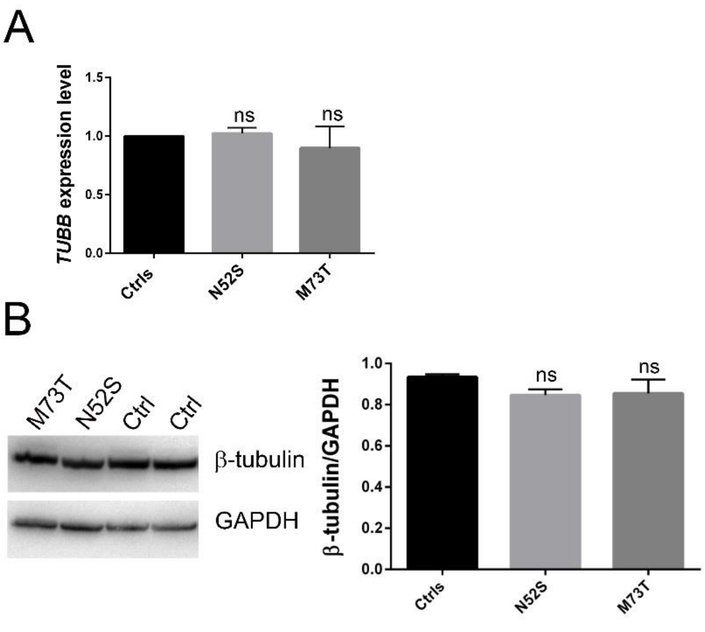

(1S**)**

Figure S1. N52S and M73T variants do not affect gene and protein expression of TUBB. To exclude that the N52S and M73T variants could elicit compensatory mechanisms that affect *TUBB* expression, we assessed by Quantitative Real-Time PCR, the levels of *TUBB* mRNA in patients' cells. As shown in Figure 1SA, there is no significant reduction in *TUBB* expression as compared to two control fibroblasts. Then, to exclude an effect of N52S and M73T substitutions on TUBB protein stability, we assessed by western blot the amount of total β-tubulin in patients' fibroblasts. The immunoblot analysis, using an antibody that recognizes multiple β-tubulin isoforms, does not any significant variation of total β-tubulin protein levels (A-B). Experiments were conducted in triplicates and values expressed as mean ± SEM.

Materials and Methods

Quantitative Real-Time PCR (qRT-PCR)

500 ηg of RNA samples was reverse transcribed with the SuperScript™ First-Strand Synthesis system and random hexamers as primers (Life Technologies, Carlsbad, CA, USA). The expression levels of TUBB was measured by qRT-PCR in an ABI PRISM 7500 Sequence Detection System (Life Technologies) using Power SYBR Green I dye chemistry (ThermoFisher Scientific, Walthman, MA, USA). Data were analyzed using the $2^{-\Delta\Delta Ct}$ method with GUSB and TBP (TATA box binding protein) as housekeeping genes, and data are shown as fold change relative to controls.

Western Blot Analysis

Fibroblasts were lysed on ice with RIPA buffer, including phosphatase and protease inhibitors (Pierce) and 20 µg of proteins were subjected to SDS PAGE on 4–12% denaturing gel and probed with the following antibodies: β-tubulin (1:1000; 1h Cell Signaling), GAPDH (1:10.000, 1h Sigma). Immunoreactive bands were detected using the Lite Ablot Extend Long Lasting Chemiluminescent substrate (Euroclone). Signals were captured by Chemi DocTM XRS 2015 (Bio-Rad Laboratories, Hercules, CA, USA) and densitometric analysis was performed using Image Lab software (Version 5.2.1, Bio-Rad Laboratories).
